# Minimally Invasive Surgery for Hirschsprung Disease: Current Practices and Future Directions

**DOI:** 10.7759/cureus.66444

**Published:** 2024-08-08

**Authors:** Mohammed Khaleel I. KH. Almadhoun, Rami Kamal Atiya Morcos, Lara Alsadoun, Syed Faqeer Hussain Bokhari, Zeeshan Ahmed, Faria Khilji, Abdul Haseeb Hasan, Danyal Bakht, Omer Abuelgasim, Mohamedalamin Alnoor Altayb Ismail

**Affiliations:** 1 Medicine and Surgery, Mutah University, Karak, JOR; 2 General Surgery, Ministry of Health Holdings, Riyadh, SAU; 3 General Surgery, Ain Shams University Hospitals, Cairo, EGY; 4 Trauma and Orthopaedics, Chelsea and Westminster Hospital, London, GBR; 5 Surgery, King Edward Medical University, Lahore, PAK; 6 Medicine and Surgery, King Edward Medical University, Lahore, PAK; 7 Internal Medicine, Tehsil Headquarter Hospital, Shakargarh, PAK; 8 Internal Medicine, Quaid-e-Azam Medical College, Bahawalpur, PAK; 9 Internal Medicine, Mayo Hospital, Lahore, PAK; 10 Internal Medicine, King Edward Medical University, Lahore, PAK; 11 Medicine and Surgery, Mayo Hospital, Lahore, PAK; 12 Rheumatology, Cork University Hospital, Cork, IRL; 13 Internal Medicine, Ibrahim Malik Teaching Hospital, Khartoum, SDN

**Keywords:** enterocolitis, surgical innovation, congenital anomalies, colorectal disorders, pediatric surgery, robotic-assisted surgery, transanal endorectal pull-through, laparoscopic surgery, minimally invasive surgery, hirschsprung disease

## Abstract

Hirschsprung disease (HD) is a congenital disorder characterized by the absence of ganglion cells in the distal colon and rectum, leading to functional obstruction and severe constipation. Over the past decades, the surgical management of HD has significantly evolved, with minimally invasive surgery (MIS) techniques revolutionizing treatment approaches. This review explores recent innovations in MIS for HD, focusing on laparoscopic, transanal endorectal pull-through (TERPT), and robotic-assisted techniques. These approaches offer numerous advantages over traditional open procedures, including reduced surgical trauma, improved cosmesis, faster recovery times, and potentially lower complication rates. Laparoscopic surgery has become widely adopted, providing excellent visualization and precise dissection. TERPT has gained popularity for short-segment disease, offering a completely transanal approach with minimal scarring. Robotic-assisted surgery represents the cutting edge, enhancing surgical precision and dexterity. The review also examines emerging technologies and future directions, such as advanced imaging techniques, artificial intelligence applications, and potential developments in tissue engineering. While MIS techniques have shown promising outcomes, challenges remain in standardizing approaches, addressing long-segment disease, and optimizing long-term functional results. The future of HD surgery lies in personalized approaches that integrate genetic and molecular profiling with advanced surgical technologies. As the field continues to evolve, comprehensive long-term studies and efforts to improve access to specialized care will be crucial to further enhancing outcomes for patients with HD.

## Introduction and background

Hirschsprung disease (HD) is a congenital disorder characterized by the absence of ganglion cells in the distal colon and rectum [1]. This absence results from a failure of neural crest cells to migrate properly during embryonic development, leading to a lack of enteric nervous system in the affected bowel segment. The aganglionic segment is unable to relax, causing functional obstruction and severe constipation [2]. HD typically affects the rectosigmoid region but can extend proximally to involve longer segments of the colon or, in rare cases, the entire colon (total colonic aganglionosis) [1]. The incidence of HD is estimated to be approximately 1 in 5,000 live births, with a male (4:1) predominance [3]. It is more common in certain populations and can be associated with other congenital anomalies or genetic syndromes [4]. The impact of HD on patients can be significant, with symptoms typically presenting in the neonatal period or early infancy. Affected newborns may experience delayed passage of meconium, abdominal distension, vomiting, and failure to thrive. The diagnosis of HD is confirmed through a rectal biopsy, which reveals the absence of ganglion cells in the intestinal wall. If left untreated, HD can lead to severe complications such as enterocolitis, toxic megacolon, and intestinal perforation, which can be life-threatening [5].

Traditionally, the surgical management of HD involved open procedures such as the Swenson, Duhamel, and Soave techniques [6,7]. These operations aimed to resect the aganglionic segment and bring the normally innervated bowel down to the anus. While effective, these procedures were associated with significant morbidity, including large abdominal incisions, prolonged hospital stays, and potential complications such as anastomotic leaks and pelvic nerve injuries. The advent of minimally invasive surgery (MIS) has revolutionized the treatment of HD, offering several advantages over traditional open techniques [8]. MIS approaches, including laparoscopic and transanal endorectal pull-through procedures, have demonstrated reduced surgical trauma, improved cosmesis, faster recovery times, and potentially lower rates of certain complications [8,9]. These techniques allow for magnified visualization of pelvic structures, potentially leading to more precise dissection and preservation of important nerves and structures.

The importance of minimally invasive techniques in HD management cannot be overstated. They have transformed the surgical approach, allowing for earlier interventions and potentially improved functional outcomes. MIS techniques have been shown to result in less postoperative pain, shorter hospital stays, and an earlier return to normal activities for patients. Additionally, the reduced abdominal wall trauma may lead to fewer long-term complications such as adhesions and incisional hernias [8,10]. The objectives of this review are to explore recent innovations in minimally invasive surgical techniques for HD and their implications for patient management. We aim to provide a comprehensive overview of current practices, including laparoscopic-assisted, totally transanal, and robotic-assisted approaches. Furthermore, we will examine emerging technologies and future directions in HD surgery, such as advanced imaging techniques, improvements in surgical instruments, and potential applications of artificial intelligence and machine learning in surgical planning and execution.

By critically analyzing these advancements, we hope to offer insights into the evolving landscape of HD treatment and provide guidance for surgeons and researchers in this field. This review will also address the challenges and limitations of current minimally invasive techniques, highlighting areas where further research and innovation are needed to improve outcomes for patients with HD.

## Review

Historical perspective

**Figure 1:**
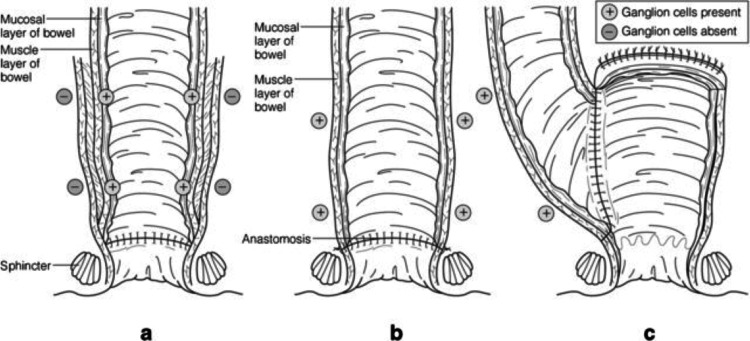
The three most commonly performed operations for Hirschsprung disease. (A) Soave procedure. In this operation, the mucosa is stripped from the underlying muscle, and the normally innervated bowel is brought through the rectal muscular “cuff” and anastomosed just above the anal sphincter. This operation was designed to prevent injury to pelvic nerves and blood vessels. (B) Swenson procedure. In this operation, the full thickness of the rectum is removed, and the normally innervated bowel is anastomosed just above the anal sphincter. (C) Duhamel procedure. In this operation, the bloodless plane behind the rectum is developed, and the normally innervated bowel is brought down and anastomosed to the back of the native aganglionic rectum just above the anal sphincter. A stapler is then used to connect the two lumens, so that the final result is a tube that consists of the aganglionic rectum anteriorly and the normally innervated bowel posteriorly. (+ = ganglionic bowel, − = aganglionic bowel) This figure is adapted with permission from the chapter on Hirschsprung disease in the textbook “Pediatric Surgery, 7th edition,” edited by Coran et al., Elsevier Saunders, 2012 [16]. Also adapted with permission from Langer et al. [17].

The surgical management of HD has undergone significant evolution since its first description by Harald Hirschsprung in 1886 [11]. Initially, the lack of understanding of the underlying pathophysiology led to inadequate treatments focused on symptom management rather than addressing the root cause of the disease. The early 20th century saw the development of various surgical techniques aimed at bypassing or removing the aganglionic segment. In 1948, Orvar Swenson and Alexander Bill introduced the first successful surgical procedure for HD, which involved resecting the aganglionic segment and performing a direct anastomosis between the ganglionic colon and the anus [12]. This technique, known as the Swenson procedure, became the preferred treatment for HD for many years. Subsequently, other open surgical techniques were developed, each with its own advantages and limitations. In 1956, Bernard Duhamel introduced a technique that involved a retro rectal pull-through, leaving the native rectum in situ as an anterior rectal wall [13,14]. The Duhamel procedure aimed to reduce the risk of pelvic nerve injury associated with the Swenson technique. In 1964, Franco Soave described the endorectal pull-through technique, which involved submucosal dissection of the rectum, potentially reducing the risk of pelvic nerve injury and cuff abscess formation [15]. These open surgical techniques, while effective, were associated with significant morbidity due to large abdominal incisions and extensive pelvic dissection (Figure 1). The transition to less invasive methods began in the late 20th century, driven by the desire to reduce surgical trauma and improve patient outcomes.

The introduction of minimally invasive techniques in HD surgery marked a significant milestone in the field. In 1995, Keith Georgeson reported the first laparoscopically assisted endorectal pull-through for HD [18]. This technique combined the benefits of minimally invasive abdominal access with the principles of the Soave procedure, allowing for smaller incisions and potentially faster recovery. Another major advancement came in 1998 when Luis De la Torre-Mondragón and José Antonio Ortega-Salgado described the totally transanal endorectal pull-through (TERPT) technique [19]. This approach eliminated the need for abdominal incisions entirely, further reducing surgical trauma and improving cosmetic outcomes. The adoption of these minimally invasive techniques faced initial challenges. Surgeons had to adapt to new instruments and develop skills in laparoscopic and transanal approaches. There were concerns about the ability to perform adequate mobilization of the colon and precise dissection in the confined pelvic space. Additionally, questions arose regarding the long-term functional outcomes of these new techniques compared to traditional open procedures. Despite these challenges, the potential benefits of minimally invasive surgery for HD drove continued innovation and refinement of techniques. Improved instrumentation, better visualization systems, and advancements in surgical training gradually overcame many of the initial obstacles.

The early 21st century saw further developments in minimally invasive HD surgery, including the introduction of single-incision laparoscopic techniques and robotic-assisted procedures [20,21]. These advancements aimed to further reduce surgical trauma and enhance the precision of dissection, particularly in complex cases involving long-segment disease or redo procedures. The historical progression of surgical techniques for HD reflects a constant pursuit of improved patient outcomes, reduced morbidity, and enhanced quality of life for those affected by this challenging condition. As we move forward, the field continues to evolve, building upon the foundational principles established by pioneers in HD surgery while embracing new technologies and approaches to further refine patient care.

Minimally invasive surgery

Minimally invasive surgery (MIS) has revolutionized the treatment of HD, offering significant advantages over traditional open procedures. The current landscape of MIS for HD encompasses several techniques, each with its own unique benefits and applications. Laparoscopic surgery has emerged as a widely adopted approach, providing reduced recovery time and minimal scarring while maintaining efficacy. The TERPT technique has gained popularity, particularly for short-segment disease, due to its minimally invasive nature and excellent outcomes [22,23]. Robotic-assisted surgery represents the cutting-edge technology in this field, offering enhanced precision and visualization during complex procedures [21]. Single-incision laparoscopic surgery (SILS) further pushes the boundaries of minimally invasive techniques, aiming to reduce surgical trauma even further [24]. These approaches have collectively transformed the management of HD, improving patient outcomes and quality of life. However, each technique has its own learning curve, specific indications, and potential limitations that warrant careful consideration in patient selection and surgical planning.

Laparoscopic surgery

Laparoscopic surgery has emerged as a cornerstone in MIS for HD, offering significant advantages over traditional open procedures. The laparoscopic approach for HD typically involves a multi-step procedure. The surgery begins with the placement of small incisions in the abdominal wall, usually ranging from 3 to 5 mm in size, through which trocars are inserted. A camera and specialized laparoscopic instruments are then introduced through these ports [18,25,26]. The procedure generally follows these key steps: (1) Diagnostic laparoscopy to confirm the transition zone and assess the bowel. (2) Mobilization of the colon, starting from the sigmoid and progressing distally to the transition zone. (3) Identification and preservation of vital structures, including the ureters and pelvic nerves. (4) Transection of the mesentery to allow for adequate length of the pull-through segment. (5) Creation of an endorectal muscular cuff, typically starting about 0.5-1 cm above the dentate line. (6) Transanal pull-through of the mobilized colon and anastomosis with the anorectal mucosa. The transition zone is identified through visual inspection and, in some cases, intraoperative frozen section biopsies. The extent of resection is determined based on the presence of ganglion cells, ensuring complete removal of the aganglionic segment.

Laparoscopic surgery for HD offers several significant benefits over traditional surgical procedures. Laparoscopy causes less trauma to the abdominal wall and internal organs, leading to faster recovery rates and shorter hospital stays as compared to open surgery. The small incisions used in laparoscopic surgery result in reduced postoperative pain. This leads to a decreased need for pain medication and earlier mobilization of patients [27]. The cosmetic outcome of laparoscopic surgery is also superior to that of open procedures. The small incisions heal with minimal scarring, which is particularly important for pediatric patients. For example, Liu et al. demonstrated that modified transanal Soave assisted by laparoscopy improves anal function, and quality of life, reduces anal pressure, and reduces complications in children with HD (all p < 0.05) [28]. The operation time, intraoperative blood loss, length of hospital stay, and gastrointestinal recovery time in the laparoscopic group were significantly lower than those in the modified group (p < 0.05). The postoperative anal function was rated as excellent or good in 87.10% of the laparoscopic group, compared to 68.97% in the modified group (p < 0.05). Additionally, 90.32% of patients in the laparoscopic group reported a good quality of life, compared to 74.14% in the modified group (p < 0.05). The total complication rate was also lower in the laparoscopic group (6.45%) compared to the modified group (22.41%) (p < 0.05) [28]. Similarly, Wang et al. also demonstrated that laparoscopic-assisted Soave procedures for long-segment HD demonstrated high success (100%), rapid recovery (hospital discharge in 5-7 days), and minimal complications in 106 cases over 10 years [29]. Menon et al. also reported that laparoscopic-assisted pull-through for HD in 28 pediatric patients showed a median surgery duration of 4 hours, a six-day hospital stay, and minimal complications, making it a safe and effective treatment option [30]. Laparoscopic surgery for Hirschsprung's disease has a low rate of complications such as anastomotic leak (0-5%) and a similar risk of enterocolitis (20-30%) and constipation as open surgery [8,27,31-34]. Long-term continence outcomes are generally good, and the risk of adhesive small bowel obstruction is reduced [35]. Success is influenced by surgeon experience and patient selection.

Transanal endorectal pull-through

Transanal endorectal pull-through (TERPT) has emerged as a significant advancement in the minimally invasive treatment of HD, particularly for patients with short-segment aganglionosis. This technique, first described by De la Torre-Mondragón and Ortega-Salgado in 1998, has gained widespread acceptance due to its minimally invasive nature and excellent outcomes [19]. The TERPT procedure is typically performed entirely through the anus without the need for abdominal incisions. The key steps of the procedure include (1) placement of stay sutures at the dentate line for retraction. (2) Submucosal dissection starts 0.5-1 cm above the dentate line, creating a muscular cuff. (3) Circumferential full-thickness incision of the rectal wall above the peritoneal reflection. (4) Mobilization of the colon by continuing the dissection proximally. (5) Identification of the transition zone and resection of the aganglionic segment. (6) Pull-through of the ganglionic bowel and coloanal anastomosis [36].

Several innovations have significantly refined the TERPT technique, enhancing its effectiveness and outcomes. One such advancement is the laparoscopic-assisted TERPT, which integrates laparoscopy for mobilizing the colon with the transanal approach, proving particularly beneficial in cases involving longer aganglionic segments [37]. Another notable innovation is the single-stage TERPT, performed without the necessity of a prior colostomy, which has demonstrated promising results in selected patients by reducing the requirement for multiple surgeries [22,38]. Additionally, modifications like the use of a shorter muscular cuff or a partial thickness cuff have been proposed by some surgeons to potentially diminish postoperative obstructive symptoms [39,40].

Patient selection is a critical factor in the success of TERPT. This surgical technique is most commonly employed for short-segment HD, particularly in cases of rectosigmoid aganglionosis [41]. The outcomes of TERPT have been widely studied and generally show several advantages. Operative time is usually shorter in comparison to open or laparoscopic approaches, leading to a quicker surgical process. Hospital stays are also typically shorter, with many centers reporting that patients can be discharged within 2-3 days after surgery [22,26,36,42]. Postoperative pain is notably reduced due to the absence of abdominal incisions, which is a significant benefit for patient comfort and recovery. While rates of enterocolitis post-surgery can vary, they are generally comparable to other techniques [26,36,42,43]. Gandhi et al. reported that the median age of children selected for TERPT surgery was nine months, with a median weight of 7.5 kg. The transition zone was located at the rectosigmoid level in eight patients (66.6%) and the sigmoid colon in four patients (33.3%). The mean length of the muscle cuff was 3 cm, and the mean length of the resected bowel was 25 cm. The median operative time was 105 minutes, and the mean hospital stay was eight days. Postoperative complications included perianal excoriation in two patients and enterocolitis in one patient, but there were no cases of cuff abscess, anastomotic leak, or stricture. Stool frequency averaged six to ten times a day initially, reducing to two to three times a day by three months postoperatively. Follow-up showed no instances of fecal soiling or constipation [44]. Overall, TERPT is a beneficial technique with positive outcomes when appropriate patient selection is applied.

TERPT offers several advantages over traditional pull-through methods for colorectal procedures. One of the primary benefits is the avoidance of abdominal incisions, which not only results in improved cosmetic outcomes but also potentially reduces postoperative pain for patients. Furthermore, TERPT preserves pelvic structures by allowing for better visualization and careful handling of pelvic nerves and other structures, thereby reducing the likelihood of damage during surgery. Another significant advantage is the reduced risk of intra-abdominal adhesions, as the lack of abdominal surgery minimizes the formation of scar tissue that can cause complications. Additionally, in selected cases, TERPT can be performed as a single-stage repair, eliminating the need for a prior colostomy and thus reducing the necessity for multiple surgeries [38]. The technique also offers improved visualization of the dentate line, allowing surgeons to place the anastomosis more precisely, which may enhance functional outcomes. As a result, patients often experience shorter hospital stays and a faster return to normal activities.

However, despite its advantages, TERPT does have some limitations. One of the main challenges is the learning curve associated with the technique, as it requires specific skills and experience that may not be readily available in all surgical settings. There is also a potential risk of missing the true transition zone, particularly in cases where laparoscopic assistance is not used, which can lead to incomplete treatment [45]. Therefore, while TERPT presents significant benefits, it is essential to consider these limitations and the specific circumstances of each patient when determining the appropriate surgical approach.

Robotic-assisted surgery

Robotic-assisted surgery represents the cutting-edge of minimally invasive techniques in the treatment of HD. This advanced approach combines the benefits of laparoscopic surgery with enhanced precision and visualization, potentially improving surgical outcomes. Robotic-assisted surgery for HD typically utilizes the da Vinci Surgical System, although other robotic platforms are in development [46-48]. The key components include a console where surgeons operate via hand controls and foot pedals, a patient-side cart housing robotic arms for surgical instruments, a high-definition 3D vision system, and wristed instruments offering enhanced dexterity owing to increased angle of articulation. The procedural steps for robotic-assisted pull-through in HD involve patient positioning, port placement for robotic arms, system docking, identification of the transition zone (aided by intraoperative biopsies), colon mobilization, creation of an endorectal muscular cuff transanally, and the crucial transanal pull-through and anastomosis [46]. Some centers have described hybrid approaches, using robotic assistance for the abdominal portion and transitioning to a transanal approach for the pull-through [49,50].

Robotic-assisted surgery presents a range of benefits compared to conventional laparoscopic techniques, primarily revolving around enhanced precision and improved visualization. First, the robotic system effectively filters out hand tremors and scales surgeon movements, resulting in remarkably precise actions during procedures such as dissection and suturing. Second, the incorporation of a 3D, high-definition vision system offers superior depth perception over traditional laparoscopy, aiding surgeons in navigating anatomical structures more accurately [51]. Moreover, the wristed instruments of robotic systems provide increased dexterity and a broader range of motion, particularly advantageous in delicate procedures within confined spaces like the pelvic area. Additionally, the ergonomic design of the seated console reduces surgeon fatigue, which is beneficial during lengthy operations. Looking forward, robotic technology holds potential for telesurgery, allowing surgeons to operate remotely, although this capability is not yet widely adopted. Lastly, these systems often include features that enhance teaching opportunities, enabling experienced surgeons to mentor and monitor less-seasoned colleagues effectively.

While robotic-assisted surgery for HD is still relatively new, emerging evidence suggests promising outcomes. In terms of effectiveness, initial studies have demonstrated favorable operative outcomes, showcasing successful completion of robotic-assisted pull-through procedures with conversion rates to open surgery either comparable to or lower than those seen with conventional laparoscopy [49,50,52]. Moreover, research suggests a potentially shorter learning curve for robotic surgery compared to traditional laparoscopic techniques, attributed to the intuitive controls and enhanced visualization offered by robotic systems [53]. Early reports also indicate functional outcomes such as bowel function and continence following robotic surgery are on par with outcomes from other minimally invasive approaches, although comprehensive long-term data are still awaited. Regarding safety, early findings suggest complication rates associated with robotic-assisted surgery are similar to or lower than those of conventional laparoscopy, although larger-scale studies are necessary to validate these observations. Specific complications reported align with those seen in other minimally invasive procedures, including anastomotic leaks, enterocolitis, and constipation [27,46]. While rates appear comparable across different techniques, additional data is crucial for a more precise assessment. Notably, concerns have been raised about potential increased port-site complications, particularly hernias, attributable to the larger port sizes required for accommodating robotic instruments. As research continues to evolve, ongoing investigation and clinical experience will be essential to fully understand the comparative safety and effectiveness of robotic-assisted surgery for HD.

Robotic surgery presents several challenges and considerations that impact its adoption and effectiveness in healthcare settings. First, cost remains a significant barrier due to the high initial investment and ongoing maintenance expenses associated with robotic systems [54]. This financial burden can deter many hospitals and clinics from integrating this technology into routine practice. Second, there is evidence suggesting longer operative times, especially during the initial learning curve for surgeons adopting robotic techniques [55]. This factor not only affects efficiency but also patient outcomes and operating room utilization. Another critical issue is the lack of tactile feedback in current robotic systems, which can potentially compromise surgical precision and the ability to detect tissue characteristics. Surgeons often rely on visual cues alone, which may not fully substitute for the sensory feedback provided by traditional surgery. Moreover, the large size of existing robotic equipment poses challenges in settings like pediatric operating rooms, where space is limited. This limitation restricts the accessibility of robotic surgery for certain patient populations. Additionally, due to its relatively recent introduction, there is a paucity of long-term outcome data comparing robotic surgery with conventional methods. The ongoing accumulation of such data is crucial for assessing the durability and efficacy of robotic-assisted procedures over time. These challenges underscore the need for continued research, technological advancements, and cost-effectiveness analyses to optimize the integration and outcomes of robotic surgery in clinical practice. As technology advances and more surgeons gain experience with robotic systems, it is likely that robotic-assisted surgery will play an increasingly important role in the management of this condition. Future developments, including smaller instruments and platforms specifically designed for pediatric use, may further enhance the applicability of this technique in the treatment of HD.

Future directions

The field of MIS for HD continues to evolve rapidly, with several promising directions for future advancement. These developments aim to further improve surgical outcomes, reduce complications, and enhance patient quality of life. The integration of advanced imaging techniques with surgical navigation systems holds great promise for HD surgery. Intraoperative near-infrared fluorescence imaging, for instance, could aid in more precise identification of the transition zone and critical structures [56]. This technology, when combined with augmented reality systems, could provide surgeons with real-time, three-dimensional visualizations of the patient's anatomy superimposed on the surgical field [57,58]. Such advancements could potentially reduce the risk of incomplete resection and improve the preservation of important structures like pelvic nerves.

The application of artificial intelligence (AI) and machine learning (ML) in surgical planning and execution is an exciting frontier. These technologies could assist in preoperative risk assessment, helping to optimize patient selection for different minimally invasive approaches. During surgery, AI-powered systems could provide real-time analysis of tissue characteristics, potentially improving the accuracy of transition zone identification [59]. Post-operatively, ML algorithms could be employed to predict and prevent complications like enterocolitis, allowing for more personalized patient management. While robotic-assisted surgery for HD is still in its early stages, future developments in robotic technology are likely to address current limitations. Smaller, more versatile robotic platforms designed specifically for pediatric patients could overcome space constraints in pediatric operating rooms. The development of haptic feedback systems for robotic instruments could restore the tactile sensation currently lacking in robotic surgery, potentially improving surgical precision and tissue handling [60].

The trend towards even less invasive approaches is likely to continue. Refinements in single-port laparoscopic techniques could further reduce surgical trauma and improve cosmetic outcomes. The concept of Natural Orifice Transluminal Endoscopic Surgery (NOTES), where surgery is performed entirely through natural orifices without external incisions, could potentially be applied to HD surgery, particularly for short-segment disease [20,61]. While technically challenging, such approaches could offer significant benefits in terms of postoperative pain and recovery time. Advances in tissue engineering could revolutionize the treatment of HD. Research into the development of engineered neural crest cells, or enteric neurons, could potentially offer new treatment modalities. These engineered tissues could be used to repopulate aganglionic segments, either as a standalone treatment or in conjunction with minimally invasive surgical techniques [62,63]. While still in the early experimental stages, such approaches hold the potential to dramatically alter the surgical management of HD.

The development of more advanced laparoscopic and endoscopic instruments specifically designed for pediatric patients could enhance the precision and safety of minimally invasive HD surgery. Novel energy devices that allow for more precise dissection and improved hemostasis in the confined pelvic space could reduce operative times and complication rates. The implementation of ERAS protocols tailored specifically for pediatric patients undergoing minimally invasive HD surgery could further improve postoperative outcomes. These protocols, which may include optimized pain management strategies, early feeding, and mobilization, could potentially reduce hospital stays and enhance recovery [64]. As minimally invasive techniques for HD become more established, there is a pressing need for long-term follow-up studies. These studies should focus not only on surgical outcomes but also on functional results, quality of life, and psychosocial impacts. Such data will be crucial in refining surgical techniques and optimizing long-term patient management.

The future of HD surgery is likely to become increasingly personalized. Genetic and molecular profiling of patients could help in tailoring surgical approaches to individual patients. This could include decisions on the extent of resection, the choice between different minimally invasive techniques, and personalized postoperative management strategies. Advancements in robotic technology and telecommunications could pave the way for telesurgery in HD management. This could allow expert surgeons to perform or assist in procedures remotely, potentially improving access to specialized care in underserved areas [51]. Additionally, remote proctoring systems could enhance surgical training and standardize techniques across different centers.

## Conclusions

The field of minimally invasive surgery for Hirschsprung disease has witnessed remarkable advancements, significantly improving patient outcomes and quality of life. Laparoscopic, transanal, and robotic-assisted techniques have demonstrated their efficacy in reducing surgical trauma, enhancing recovery, and maintaining long-term functional results. However, challenges remain, particularly in terms of standardizing techniques, addressing long-segment disease, and optimizing long-term outcomes. The future of HD surgery lies in the integration of cutting-edge technologies such as advanced imaging, artificial intelligence, and robotic systems. These innovations promise to enhance surgical precision, improve decision-making, and potentially lead to novel treatment modalities. The trend towards increasingly personalized approaches based on genetic and molecular profiling may further refine patient management strategies. As the field evolves, there is a critical need for comprehensive, long-term studies to evaluate functional outcomes and quality of life. Additionally, efforts to improve access to specialized care through telesurgery and standardized training programs will be crucial. By continuing to innovate and refine minimally invasive techniques, the surgical management of Hirschsprung disease is poised to offer even better outcomes for patients in the years to come.
